# Reference phantom selection in pediatric computed tomography using data from a large, multicenter registry

**DOI:** 10.1007/s00247-021-05227-0

**Published:** 2021-12-06

**Authors:** Philip W. Chu, Sophronia Yu, Yifei Wang, J. Anthony Seibert, Luisa F. Cervantes, Nima Kasraie, Cameron A. Chu, Rebecca Smith-Bindman

**Affiliations:** 1 Department of Epidemiology & Biostatistics, University of California San Francisco, 550 16th St., Box 0560, San Francisco, CA 94143 USA; 2 Chan Zuckerberg Biohub, San Francisco, CA USA; 3 Department of Radiology, University of California Davis Health, Sacramento, CA USA; 4 Department of Radiology, Nicklaus Children’s Hospital, Miami, FL USA; 5 Department of Radiology, UT Southwestern Medical Center, Dallas, TX USA; 6 Department of Obstetrics, Gynecology and Reproductive Sciences, University of California San Francisco, San Francisco, CA USA; 7 Philip R. Lee Institute for Health Policy Studies, University of California San Francisco, San Francisco, CA USA

**Keywords:** Children, Computed tomography, Phantom, Radiation dose, Reference phantom, Registry

## Abstract

**Background:**

Radiation dose metrics vary by the calibration reference phantom used to report doses. By convention, 16-cm diameter cylindrical polymethyl-methacyrlate phantoms are used for head imaging and 32-cm diameter phantoms are used for body imaging in adults. Actual usage patterns in children remain under-documented.

**Objective:**

This study uses the University of California San Francisco International CT Dose Registry to describe phantom selection in children by patient age, body region and scanner manufacturer, and the consequent impact on radiation doses.

**Materials and methods:**

For 106,837 pediatric computed tomography (CT) exams collected between Jan. 1, 2015, and Nov. 2, 2020, in children up to 17 years of age from 118 hospitals and imaging facilities, we describe reference phantom use patterns by body region, age and manufacturer, and median and 75th-percentile dose–length product (DLP) and volume CT dose index (CTDI_vol_) doses when using 16-cm vs. 32-cm phantoms.

**Results:**

There was relatively consistent phantom selection by body region. Overall, 98.0% of brain and skull examinations referenced 16-cm phantoms, and 95.7% of chest, 94.4% of abdomen and 100% of cervical-spine examinations referenced 32-cm phantoms. Only GE deviated from this practice, reporting chest and abdomen scans using 16-cm phantoms with some frequency in children up to 10 years of age. DLP and CTDI_vol_ values from 16-cm phantom-referenced scans were 2–3 times higher than 32-cm phantom-referenced scans.

**Conclusion:**

**Reference phantom selection is highly consistent, with a small but significant number of abdomen and chest scans (~5%) using 16-cm phantoms in younger children, which produces DLP values approximately twice as high as exams referenced to 32-cm phantoms:**

**Supplementary Information:**

The online version contains supplementary material available at 10.1007/s00247-021-05227-0.

## Introduction

The rapid rise over the last few decades in computed tomography (CT) imaging and consequent population exposure to ionizing radiation, a known carcinogen, have raised concerns about the levels and variability of radiation doses across patients, institutions and countries, as well as the need for dose optimization [1–8]. Diverse organizations and campaigns, such as Choosing Wisely and Image Gently, promote improving the safety and effective imaging care of children worldwide to optimize and reduce patient radiation dose exposures [9, 10].

Dose optimization tools like diagnostic reference levels use metrics such as the volume CT dose index (CTDI_vol_), reflecting the average dose (per slice) over the total volume scanned for the selected CT conditions of operation, and the dose–length product (DLP), reflecting the total dose imparted to the patient. While these metrics reflect scanner output and not patient absorbed dose, they correlate closely with absorbed doses and help physicians and imaging practices compare their doses to a uniform standard [11].

CTDI_vol_ values are reported directly from the scanner and must be referenced to a calibration reference phantom for reporting. By convention, 16-cm diameter cylindrical polymethyl-methacyrlate phantoms are used for head imaging and 32-cm diameter phantoms are used for body imaging in adults. Accuracy (validity) of the estimated dose to reflect the true patient absorbed dose depends on the closeness of fit between the volumes of the imaged body section and the reference phantom, as well as kilovoltage peak (kVp) setting and bow-tie filter. The 32-cm body phantom corresponds to a patient with a 47-in. (~120 cm) waistline. Therefore, a dose estimate for very small patients based on a 32-cm phantom at 120 kVp will underestimate the true patient absorbed dose by approximately a factor of 2, and vice versa; a CTDI_vol_ of 8 mGy from a 16-cm phantom vs. a CTDI_vol_ of 4 mGy from a 32-cm phantom would indicate the same CT output [12, 13].

An underappreciated challenge in pediatric dosimetry concerns the choice of phantom for dose reporting, as pediatric phantom selection may be inconsistent [14]. The source of this variation may be that some manufacturers follow adult conventions, while other manufacturers choose the smaller 16-cm phantoms for reporting abdomen and chest doses in children, as this more closely reflects actual patient size [15, 16]. The reported dose will vary considerably between the two phantom sizes, even when the technical parameters are identical [17]. This inconsistency in reporting can result in patient distress and confusion when they undergo scans on machines with different reporting conventions [18].

Several investigators have created ad hoc corrections, for example suggesting that CTDI_vol_ and DLP values estimated from 32-cm diameter phantoms should be multiplied by a factor of 2 to obtain “correct” values in pediatric body scans [19]. This problem is not only important when understanding an individual patient’s dose, but also when trying to optimize protocols because the applicability of a benchmark will vary depending on what phantom was used. Some pediatric reference values have been explicitly reported using only one specific size reference phantom, but unless dose comparisons use the same size phantom, it is easy to unknowingly introduce errors [20]. Similarly, the Alliance for Radiation Safety in Pediatric Imaging created conversion factors for normalizing CTDI_vol_ and DLP to patient size to estimate actual absorbed doses and specified that these values be consistently calculated with the 32-cm phantom [21].

Despite recognition of the importance of phantom selection in pediatric dosimetry, we lack representative data on what phantoms are used in actual practice, how these selections vary by manufacturer, and how the reported doses vary by phantom size in actual practice. Using data from a large multicenter CT dose registry, this study describes variations in practice and differences in estimated doses that result from the differential use of 16-cm (head) and 32-cm (body) phantoms in young patients.

## Materials and methods

### Registry

The University of California San Francisco (UCSF) International CT Dose Registry includes 6.65 million CT exams assembled from across 160 hospital and imaging facilities [6, 7]. The registry was created with funding from the University of California Office of the President, the Centers for Disease Control and Prevention, the National Institutes of Health and the Patient Centered Outcomes Research Institute, and includes data from health care institutions that used Radimetrics Radiation Dose Management Solution (Bayer HealthCare, Whippany, NJ) and expressed interest in collaborating with UCSF on radiation-related research. The UCSF Institutional Review Board approved the registry study and waived informed consent. Collaborating institutions either approved the study locally or relied on UCSF approval.

### Study population

We included 106,837 pediatric diagnostic CT examinations obtained in 118 U.S. facilities for children under 18 years of age performed between Jan. 1, 2015, and Nov. 2, 2020, that included imaging of the head, cervical spine (c-spine), chest, or abdomen and pelvis (abdomen). We divided head scans into brain and skull imaging (including sinus, facial bones and temporal bones); neck and c-spine exams are included in a single category. These body regions reflect 87% of all exams during the study period. We excluded CTs that included insufficient numbers for analysis or that covered multiple body parts (*n*=15,849 or 13% of all scans), or those performed as part of radiation oncology guidance, surgical or interventional procedures, combined positron emission tomography (PET)-CT and single photon emission CT (SPECT) imaging.

### Manufacturers

We included scans from four manufacturers: Canon Medical Systems Corporation (including Toshiba; Ōtawara, Tochigi, Japan), GE Healthcare (Chicago, Illinois), Philips (Koninklijke Philips N.V., Amsterdam, The Netherlands) and Siemens (Siemens Healthcare, Erlangen, Germany). The sample includes 41 unique scanner models (Canon/Toshiba: 5, GE: 18, Philips: 6, Siemens: 12) and 247 individual scanners.

### Variables

We report DLP, which reflects the total scanner emitted radiation, defined as the product of CTDI_vol_ and the scan length, reflecting the total radiation output received by the patient for a CT scan and measured in mGy·cm. Each dose metric is referenced to a 16-cm or 32-cm phantom. Results are shown for complete CT examinations including all irradiating events (excluding scouts, localizers and boluses). A CT examination including a scan with and a scan without contrast is considered a single examination. Exam-level DLP is calculated as the sum of all constituent series-level DLP values. For simplicity, we excluded multiphase examinations that were referenced to more than one phantom (*n*=7,204). We categorized patients into the five mutually exclusive age groups used by the Leapfrog Group [22]: <1 year, 1–4 years, 5–9 years, 10–14 years, 15–17 years.

### Statistical analysis

For each body region, we report the number and percent of examinations that used 16-cm and 32-cm phantoms, stratified by body region, patient age and manufacturer. We calculated the number and proportion of exams using the “expected” phantom, based on predominate usage patterns across all manufacturers (16 cm for brain and skull, 32 cm for chest, abdomen and c-spine) by body region, age group and scanner manufacturer.

We calculated the median and 75th percentiles for each dose metric, stratified by body region, patient age and manufacturer, and calculated the relative median dose (i.e. ratio) between phantom sizes (16 cm vs. 32 cm) to measure the magnitude of difference due to reference phantom selection when there were at least 5 CT examinations performed by age and body region using each phantom. The Radimetrics dose tracking platform was employed to extract all patient, scanner and exam variables (see [6, 7] for details), and SAS (version 9.3; SAS Institute, Cary, NC) and R (version 3.6.3; R Foundation for Statistical Computing, Vienna, Austria) were used for all analyses.

## Results

Overall, 54.6% of the exams were comprised of males, 59.2% of exams used 16-cm phantoms and the most common body region imaged was the head, including the brain (*n*=48,680, 45.6%) and skull (*n*=12,929, 12.1%). A total of 44.1% of exams were performed at pediatric-specific hospitals (Table 1). Across all body regions, the number of scans generally increased with age (Table 2).

**Table 1:** Number of computed tomography examinations by patient factors, manufacturer and phantom

	*n*	Percent
Total	106,837	100.0
Sex		
Female	48,473	45.4
Male	58,364	54.6
Age group		
<1 years	8,433	7.9
1–4 years	16,316	15.3
5–9 years	20,657	19.3
10–14 years	28,502	26.7
15–17 years	32,929	30.8
Body region		
Brain	48,680	45.6
Skull	12,929	12.1
Cervical spine	9,722	9.1
Chest	9,459	8.9
Abdomen	26,047	24.4
Facility		
Pediatric hospital	47,105	44.1
Other hospital	59,732	55.9
Manufacturer		
Canon	11,188	10.5
GE	38,395	35.9
Philips	5,218	4.9
Siemens	52,036	48.7
Phantom		
16-cm	63,239	59.2
32-cm	43,598	40.8

**Table 2:** Number of computed tomography examinations by body region and patient age, indicating the percent of brain and skull exams reported using the 16-cm phantom and the percent of cervical spine, chest and abdomen exams reported using the 32-cm phantom

Age group	Brain		Skull		Cervical spine		Chest		Abdomen	
	*n*	Percent 16-cm phantom	*n*	Percent 16-cm phantom	*n*	Percent 32-cm phantom	*n*	Percent 32-cm phantom	*n*	Percent 32-cm phantom
<1 year	6,218	99.9%	438	99.3%	245	100%	997	92.5%	535	76.8%
1–4 years	9,441	99.9%	1,673	99.2%	1,490	100%	1,435	88.8%	2,277	79.7%
5–9 years	9,040	99.8%	3,295	99.0%	1,780	100%	1,538	92.2%	5,004	88.0%
10–14 years	11,673	99.7%	3,905	99.1%	2,674	100%	2,335	98.1%	7,915	97.1%
15–17 years	12,308	99.8%	3,618	98.2%	3,533	100%	3,154	99.7%	10,316	99.5%
All ages	48,680	99.8%	12,929	98.8%	9,722	100%	9,459	95.7%	26,047	94.4%

Phantom selection varied by body region, and for most patients the phantom choice was the same as in adults (Table 2). The 16-cm phantom was used in more than 98.0% of examinations for brain and skull CT examinations regardless of patient age. The 32-cm phantom was used for most chest examinations (95.7%) and abdomen examinations (94.4%), and use of the 32-cm phantom increased with increasing patient age. The 32-cm phantom was used for 100% of c-spine CT examinations.

**Fig. 1:**
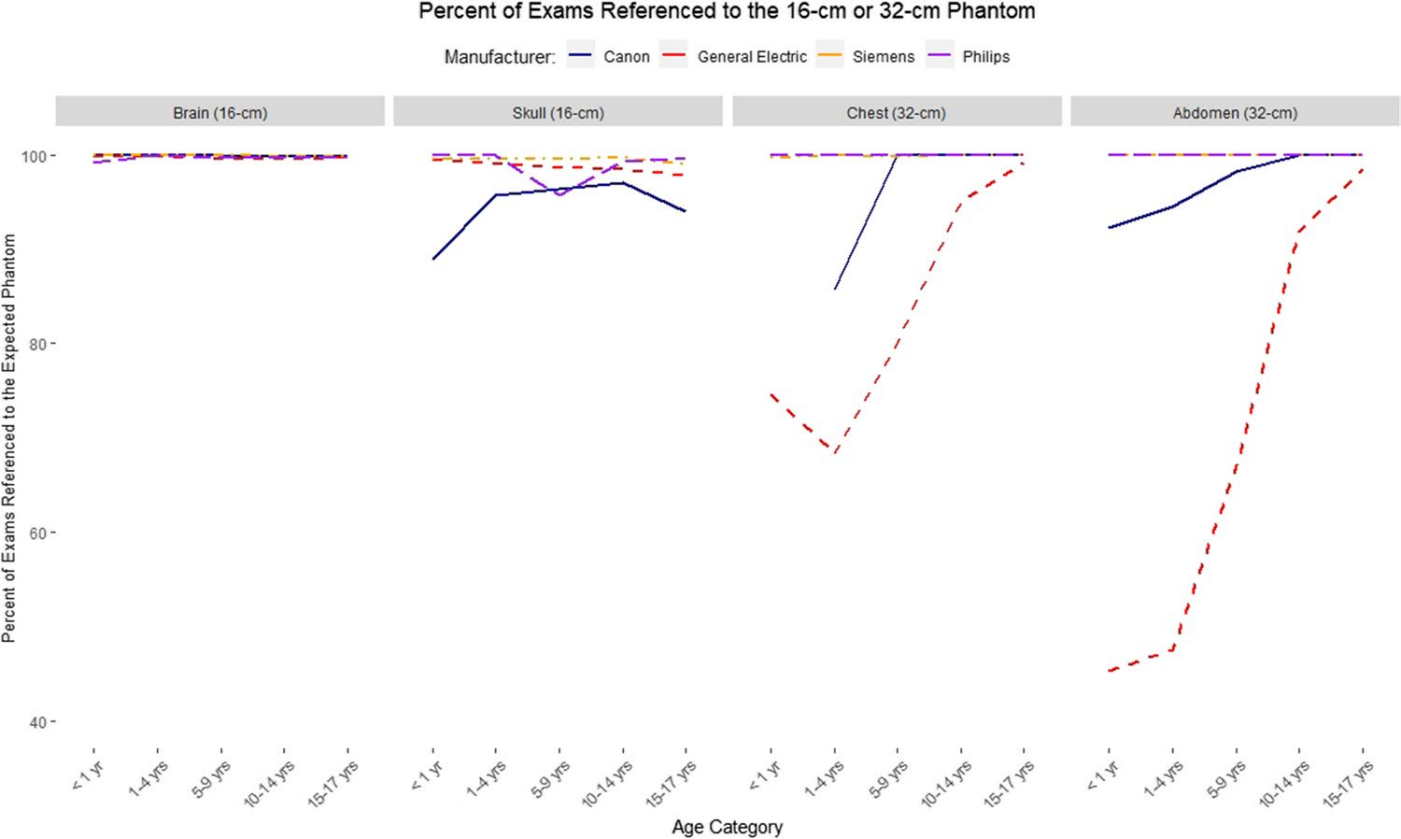
Percent of brain and skull exams referenced to the 16-cm phantom and percent of chest and abdomen exams referenced to the 32-cm phantom, by age group and manufacturer

We observed consistent use of the 16-cm phantom for brain and skull imaging across manufacturers with few exceptions, while greater differences in phantom selection by manufacturer were observed for chest and abdomen CT examinations (Table 3). Philips and Siemens used the 32-cm phantom in more than 99% of children for both chest and abdomen CT. For chest CT, Canon used the 32-cm phantom uniformly above age 5, and GE used the 32-cm phantom in more than 95% of children above age 10; in the younger age groups, GE used the 32-cm phantom in 74.7–80.0%. For abdomen CT, GE used the 16-cm phantom frequently in children up to age 10. There is a clear relationship with age in the use of the 32-cm phantom for reporting abdomen and chest CT (Fig. 1). For example, for GE, use of the 32-cm phantom ranges from 45.3% of children <1 year old to 98.5% of children 15–17 years old; for Canon, use of the 32-cm phantom ranges from 92.3% of children <1 year old to 100% of children 15–17 years old.

**Table 3:** Number of computed tomography (CT) examinations by age, body region and manufacturer reported using the expected phantom (for brain and skull CT: 16-cm phantom, for cervical spine, chest and abdomen: 32-cm phantom)

		Canon		GE		Philips		Siemens	
		*n*	Percent using expected phantom	*n*	Percent using expected phantom	*n*	Percent using expected phantom	*n*	Percent using expected phantom
Brain	<1 y	395	100.%	2,143	99.8%	113	99.1%	3,567	99.9%
	1–4 y	1,005	100%	3,528	99.8%	409	100%	4,499	99.9%
	5–9 y	960	99.9%	3,523	99.6%	398	99.8%	4,159	99.9%
	10–14 y	1,479	99.8%	4,262	99.6%	811	99.8%	5,121	99.8%
	15–17 y	1,876	100%	4,577	99.7%	1,139	99.7%	4,716	99.8%
Skull	<1 y	9	88.9%	204	99.5%	7	100%	218	99.5%
	1–4 y	71	95.8%	743	99.1%	40	100%	819	99.5%
	5–9 y	139	96.4%	1,281	98.6%	47	95.7%	1,828	99.6%
	10–14 y	237	97.1%	1,276	98.4%	138	99.3%	2,254	99.7%
	15–17 y	349	94.0%	1,236	97.8%	231	99.6%	1,802	99.1%
Cervical spine	<1 y	11	100%	72	100%	1	100%	161	100%
	1–4 y	69	100%	499	100%	23	100%	899	100%
	5–9 y	101	100%	609	100%	31	100%	1,039	100%
	10–14 y	274	100%	705	100%	170	100%	1,525	100%
	15–17 y	535	100%	794	100%	293	100%	1,911	100%
Chest	<1 y	1	0%	284	74.7%	5	100%	707	99.7%
	1–4 y	14	85.7%	503	68.4%	1	100%	917	100%
	5–9 y	35	100%	596	80.0%	7	100%	900	99.9%
	10–14 y	69	100%	899	95.1%	10	100%	1,357	100%
	15–17 y	255	100%	1,248	99.1%	44	100%	1,607	100%
Abdomen	<1 y	13	92.3%	225	45.3%	6	100%	291	100%
	1–4 y	163	94.5%	863	47.5%	22	100%	1,229	99.9%
	5–9 y	693	98.3%	1,784	67.0%	144	100%	2,383	100%
	10–14 y	992	99.9%	2,787	91.9%	384	100%	3,752	100%
	15–17 y	1,443	100%	3,754	98.5%	744	100%	4,375	100%

*y* years

Most of the CT examinations are reported using a consistent phantom choice. Nonetheless, 0.4% of head scans (*n*=243) used 32-cm phantoms and 5.2% of chest and abdomen scans (*n*=1,877) used 16-cm phantoms. The use of 32-cm phantoms for head/skull scans is difficult to explain, though we suspect the use of “body” protocols could play a role. The use of 16-cm phantoms in chest and abdomen scans, on the other hand, could indicate intentional efforts to select a best size match, or manufacturer-specific rules related to scanning parameters such as field of view.

The use of different phantoms has a large impact on reported dose metrics. The median DLP by body region, patient age and manufacturer reported when using each phantom is shown in Table 4. Note that we omitted all c-spine and all Philips combinations from the table because none had the minimum number of five scans of each phantom size to allow comparison. The relative median DLP is approximately twofold higher when using 16-cm phantom (range: 0.7–4.9). While DLP generally increases with advancing age in the pediatric population (not necessarily in adults), these data show that the relative DLP (between 16-cm and 32-cm phantoms) generally declines with advancing age, though inconsistently. For example, the relative dose for chest exams in GE scanners actually increases from 2.8 in patients <1 year old to 4.9 in patients 1–4 years old, before decreasing thereafter for reasons we cannot explain. Results are similar when comparing the 75th percentiles of DLP and relative DLP, with an average 1.9-fold higher dose (range: 0.8–6.0) when reported using the 16-cm phantom (Online Supplementary Material 1).

**Table 4:** Median dose–length product (DLP) by body region, patient age, manufacturer and phantom, and relative DLP comparing 16-cm with 32-cm phantoms

Body region	Age category	DLP in mGy·cm								
		Canon			GE			Siemens		
		Phantom		Relative dose	Phantom		Relative dose	Phantom		Relative dose
		32-cm	16-cm		32-cm	16-cm		32-cm	16-cm	
Brain	<1 y									
	1–4 y				126	309	2.5			
	5–9 y				311	451	1.4			
	10–14 y				257	502	2.0	320	515	1.6
	15–17 y				318	641	2.0	291	636	2.2
Skull	<1 y									
	1–4 y				78	221	2.8			
	5–9 y	160	188	1.2	136	253	1.9	41	174	4.3
	10–14 y	241	390	1.6	150	284	1.9	105	221	2.1
	15–17 y	481	520	1.1	494	361	0.7	297	253	0.9
Chest	<1 y				25	69	2.8			
	1–4 y				41	198	4.9			
	5–9 y				56	228	4.1			
	10–14 y				144	570	4.0			
	15–17 y				225	288	1.3			
Abdomen	<1 y				43	72	1.7			
	1–4 y	82	148	1.8	73	141	1.9			
	5–9 y	140	198	1.4	168	192	1.1			
	10–14 y				289	357	1.2			
	15–17 y				336	585	1.7			

Values are not shown when there were fewer than 5 computed tomography examinations performed by age and body region using each phantom (numbers of scans can be derived from the *n* and percent values of Table 3). *y* years

Table 5 shows the same comparisons for CTDI_vol_, which partially removes scan length as a confounding factor. In almost all cases, the comparable ratios of relative dose exceed the values for DLP (Table 4). Results for the 75th percentiles of CTDI_vol_ are similar to the medians, with relative doses two- to threefold higher (range: 1.0–5.5) when reported using the 16-cm phantom (Online Supplementary Material 2).

**Table 5:** Median volume computed tomography (CT) dose index (CTDI_vol_) by body region, patient age, manufacturer and phantom, and relative CTDI_vol_ comparing 16-cm with 32-cm phantoms

Body region	Age category	CTDI_vol_ in mGy								
		Canon			GE			Siemens		
		Phantom		Relative dose	Phantom		Relative dose	Phantom		Relative dose
		32-cm	16-cm		32-cm	16-cm		32-cm	16-cm	
Brain	<1 y									
	1–4 y				5	17	3.5			
	5–9 y				10	27	2.8			
	10–14 y				9	31	3.3	12	29	2.4
	15–17 y				11	37	3.3	8	35	4.1
Skull	< 1 y									
	1–4 y				3	17	5.8			
	5–9 y	6	11	1.8	2	18	7.9	3	11	4.4
	10–14 y	13	27	2.1	4	20	4.6	4	14	3.4
	15–17 y	17	29	1.7	13	23	1.8	11	15	1.4
Chest	<1 y				1	4	2.5			
	1–4 y				2	6	3.1			
	5–9 y				2	7	3.1			
	10–14 y				4	10	2.3			
	15–17 y				6	9	1.5			
Abdomen	<1 y				2	3	1.7			
	1–4 y	2	6	2.5	2	4	2.0			
	5–9 y	4	7	1.9	4	5	1.2			
	10–14 y				6	8	1.4			
	15–17 y				6	11	1.8			

Values are not shown when there were fewer than 5 CT examinations performed by age and body region using each phantom (numbers of scans can be derived from the *n* and percent values of Table 3). *y* years

## Discussion

Using a large multicenter CT dose registry, we report phantom selection by body region, patient age and scanner manufacturer, and its impact on reported dose. Our findings demonstrate that most scans are reported consistently: 99% of head scans are reported using the 16-cm phantom and 95% of chest and abdomen scans are reported using the 32-cm phantom. Nonetheless, the overall consistency masks notable differences in phantom selection by manufacturer, most notably that GE frequently uses the 16-cm phantom for abdomen CT in younger children. We found, as expected, that the reported DLP values are approximately twice as high when the 16-cm vs. the 32-cm phantom is selected. With growing interest in CT dose documentation, reflected in annual hospital surveys of pediatric doses performed by the Leapfrog Group [22] and regulatory requirements of radiation dose reports in the medical record [23], it is important to use consistent standards across all patients so that physicians, radiology technologists, patients and researchers can clearly and accurately understand the results and know that they were calculated consistently.

While our research highlights both similarities and differences in phantom selection by manufacturer, even consistency in reporting might not reflect best practice. For example, while all manufacturers used the 32-cm phantom for c-spine exams, because of the large difference in size between the 32-cm phantom and child (or adult) neck sizes, reported DLP values for neck scans will markedly underestimate absorbed doses unless some adjustment factor is employed. Similarly, GE frequently uses the 16-cm phantom when reporting chest and abdomen doses in small children — a sensible decision when the 16-cm phantom more closely approximates their size than the 32-cm phantom. Yet this will result in reporting of a significantly higher dose for a given child than had that child been scanned on a device that used the 32-cm phantom. The impact on a patient when they are scanned using devices that report differently can be substantial [19]. Watson and Coakley [14] reported the inconsistent rules that the manufacturers used for selection of the phantom over a decade ago.

There is a robust discussion in the radiology and medical physics literature regarding how to best estimate patient absorbed dose using scanner output combined with information on patient size in order to understand how well scan settings have been tailored to patient size [14]. As an objective way to adjust the CTDI_vol_ to a closer representation of the actual dose delivered to the patient, and hence partially correct for the mismatch between phantom and patient dimensions, size-specific dose estimates (SSDE) were developed [24, 25]. Nonetheless, how accurately reported dose will reflect patient absorbed dose when the phantom is poorly matched to patient size remains an important question because patient doses will be underestimated when 32-cm phantoms are used in smaller patients. This paper does not address this important question, but instead focuses on how the basic interpretation of CT scanner dose output is highly dependent on which phantom is used for reporting. The scanner output must be understood in terms of the actual phantom selected; on average, all else equal, the same DLP dose output will be reported approximately twofold higher if it is scaled to a 16-cm rather than a 32-cm phantom. Our purpose was to demonstrate the magnitude of typical differences in dose that may be obscured by existing pediatric reference value studies and individual clinical applications.

This study has limitations. The sample includes data filtered through a single dose-management software vendor. However, all metrics come directly from either the radiation dose structured report or from the dose report images (via optical character recognition). Consequently, this convenience sample should not affect the phantom-derived dose differences we found. These analyses are limited to 41 scanner models from 4 manufacturers. The current sample size is insufficient to stratify phantom usage patterns by type of facility (e.g., pediatric vs. adult hospital or academic vs. community setting); however, this would be an important and worthwhile area of future study. Ideally, one would stratify and determine optimal pediatric dosing by patient size rather than age, which is a relatively poor predictor of patient diameter [26]. However, actual patient size is usually missing from Digital Imaging and Communications in Medicine data, and we were not able to generate tables by patient size. Similarly, we do not report SSDE values as they are frequently missing in the Radimetrics-derived data, unlike DLP and CTDI_vol_. In addition, we did not attempt to control for kVp setting, which is known to impact conversion of CTDI_vol_ from 16-cm to 32-cm phantoms. Lastly, this paper did not explore manufacturer rules and algorithms for phantom selection, though this is an important question for future study.

## Conclusion

These analyses empirically elucidate reference phantom selection patterns by body region, patient age and scanner manufacturer, and also demonstrate the substantial differences in scanner-reported DLP that arise due to reference phantom selection in clinical studies. Without specifying or stratifying by phantom size, any reporting of aggregate DLP values unwittingly will show a weighted summary that depends on the (unspecified) mixture of scanner manufacturers, patient ages and sizes, and phantoms used. While the use of SSDE avoids some of these problems, standardization of both phantom selection and phantom reporting would improve clinical, research and monitoring applications.

## Supplementary Information

ESM 1 (DOCX 18 kb)

ESM 2 (DOCX 18 kb)
