# Supplementary material for: Reference phantom selection in pediatric computed tomography using data from a large, multicenter registry
Source: Pediatr Radiol. 2021 Dec 6;52(3):445–52. doi: 10.1007/s00247-021-05227-0 (PMC8857172; doi:10.1007/s00247-021-05227-0)
Supplement: Supplementary file 1 — (DOCX 18 kb) [file 247_2021_5227_MOESM1_ESM.docx]

**Online Supplementary Material 1** 75th percentile dose-length product (DLP) by body region, patient age, manufacturer and phantom, and relative DLP comparing 16-cm with 32-cm phantoms

|  |  | Canon | | | GE | | | Siemens | | |
| --- | --- | --- | --- | --- | --- | --- | --- | --- | --- | --- |
|  |  | DLP | |  | DLP | |  | DLP | |  |
|  |  | Phantom | |  | Phantom | |  | Phantom | |  |
| Body region | Age | 32-cm | 16-cm | Ratio | 32-cm | 16-cm | Ratio | 32-cm | 16-cm | Ratio |
| Brain | <1 y |  |  |  |  |  |  |  |  |  |
|  | 1–4 y |  |  |  | 255 | 485 | 1.9 |  |  |  |
|  | 5–9 y |  |  |  | 418 | 594 | 1.4 |  |  |  |
|  | 10–14 y |  |  |  | 430 | 753 | 1.8 | 603 | 665 | 1.1 |
|  | 15–17 y |  |  |  | 605 | 863 | 1.4 | 463 | 760 | 1.6 |
| Skull | <1 y |  |  |  |  |  |  |  |  |  |
|  | 1–4 y |  |  |  | 179 | 359 | 2.0 |  |  |  |
|  | 5–9 y | 204 | 327 | 1.6 | 204 | 417 | 2.0 | 77 | 240 | 3.1 |
|  | 10–14 y | 489 | 575 | 1.2 | 300 | 518 | 1.7 | 310 | 291 | 0.9 |
|  | 15–17 y | 577 | 629 | 1.1 | 724 | 658 | 0.9 | 419 | 337 | 0.8 |
| Chest | <1 y |  |  |  | 38 | 113 | 3.0 |  |  |  |
|  | 1–4 y |  |  |  | 56 | 338 | 6.0 |  |  |  |
|  | 5–9 y |  |  |  | 99 | 485 | 4.9 |  |  |  |
|  | 10–14 y |  |  |  | 217 | 710 | 3.3 |  |  |  |
|  | 15–17 y |  |  |  | 348 | 800 | 2.3 |  |  |  |
| Abdomen | <1 y |  |  |  | 56 | 93 | 1.7 |  |  |  |
|  | 1–4 y | 141 | 168 | 1.2 | 100 | 191 | 1.9 |  |  |  |
|  | 5–9 y | 196 | 242 | 1.2 | 240 | 235 | 1.0 |  |  |  |
|  | 10–14 y |  |  |  | 359 | 531 | 1.5 |  |  |  |
|  | 15–17 y |  |  |  | 533 | 707 | 1.3 |  |  |  |

Values are not shown when there were fewer than 5 CT examinations performed by age and body region using each phantom (numbers of scans can be derived from the *n* and percent values of Table 3). *y* years
