# Supplementary material for: Reference phantom selection in pediatric computed tomography using data from a large, multicenter registry
Source: Pediatr Radiol. 2021 Dec 6;52(3):445–52. doi: 10.1007/s00247-021-05227-0 (PMC8857172; doi:10.1007/s00247-021-05227-0)
Supplement: Supplementary file 2 — (DOCX 18 kb) [file 247_2021_5227_MOESM2_ESM.docx]

**Online Supplementary Material 2** 75th percentile volume CT dose index (CTDIvol) by body region, patient age, manufacturer and phantom, and relative CTDIvol comparing 16-cm with 32-cm phantoms

|  |  | Canon | | | GE | | | Siemens | | |
| --- | --- | --- | --- | --- | --- | --- | --- | --- | --- | --- |
|  |  | CTDIvol | |  | CTDIvol | |  | CTDIvol | |  |
|  |  | Phantom | |  | Phantom | |  | Phantom | |  |
| Body region | Age | 32-cm | 16-cm | Ratio | 32-cm | 16-cm | Ratio | 32-cm | 16-cm | Ratio |
| Brain | <1 y |  |  |  |  |  |  |  |  |  |
|  | 1–4 y |  |  |  | 8 | 29 | 3.5 |  |  |  |
|  | 5–9 y |  |  |  | 12 | 34 | 2.8 |  |  |  |
|  | 10–14 y |  |  |  | 12 | 43 | 3.5 | 14 | 36 | 2.6 |
|  | 15–17 y |  |  |  | 14 | 49 | 3.4 | 17 | 42 | 2.5 |
| Skull | <1 y |  |  |  |  |  |  |  |  |  |
|  | 1–4 y |  |  |  | 4 | 23 | 5.5 |  |  |  |
|  | 5–9 y | 12 | 29 | 2.4 | 5 | 28 | 5.3 | 3 | 18 | 5.4 |
|  | 10–14 y | 17 | 29 | 1.6 | 9 | 29 | 3.1 | 10 | 20 | 2.0 |
|  | 15–17 y | 18 | 31 | 1.7 | 19 | 32 | 1.7 | 15 | 21 | 1.4 |
| Chest | <1 y |  |  |  | 2 | 5 | 2.8 |  |  |  |
|  | 1–4 y |  |  |  | 2 | 7 | 3.0 |  |  |  |
|  | 5–9 y |  |  |  | 3 | 9 | 3.0 |  |  |  |
|  | 10–14 y |  |  |  | 6 | 10 | 1.7 |  |  |  |
|  | 15–17 y |  |  |  | 8 | 10 | 1.2 |  |  |  |
| Abdomen | <1 y |  |  |  | 2 | 3 | 1.6 |  |  |  |
|  | 1–4 y | 4 | 6 | 1.6 | 3 | 6 | 2.1 |  |  |  |
|  | 5–9 y | 5 | 9 | 1.8 | 6 | 6 | 1.0 |  |  |  |
|  | 10–14 y |  |  |  | 7 | 11 | 1.6 |  |  |  |

Values are not shown when there were fewer than 5 CT examinations performed by age and body region using each phantom (numbers of scans can be derived from the *n* and percent values of Table 3). *y* years
